# Experiences of expatriate university teachers in a health science university in Saudi Arabia-A Qualitative study

**DOI:** 10.12669/pjms.36.4.1896

**Published:** 2020

**Authors:** Uzma Asif, Nusrat Bano, Hend Al Najjar

**Affiliations:** 1 Dr. Uzma Asif, Ph.D. Department of Biochemistry, King Saud bin Abdulaziz University for Health Sciences, Jeddah, Kingdom of Saudi Arabia; 2 Dr. Nusrat Bano, Ph.D. Department of Pharmacology, King Saud bin Abdulaziz University for Health Sciences, Jeddah, Kingdom of Saudi Arabia; 3 Dr. Hend Al Najjar. Ph.D. Associate Dean, Department of Academic Affairs, King Saud bin Abdulaziz University for Health Sciences, Jeddah, Kingdom of Saudi Arabia

**Keywords:** Expatriate, Experiences, University, Teachers

## Abstract

**Background and Objective:**

A large number of university teachers in Saudi Arabia comprise of expatriates. Their experiences are unique in context of the challenges and benefits of academic expatriation. The purpose of this study was to describe the experiences of expatriate university teachers in Saudi Arabia.

**Methods:**

A qualitative descriptive design was used based on in-depth interviews with academic expatriates, recruited through snowball sampling in a Health Science University in Saudi Arabia. The study was conducted from 12 September 2019 to 20 October 2019 after IRB approval. Graneheimian inductive approach was used for content analysis of the data. Standard principles of trustworthiness were applied.

**Results:**

Three major themes emerged as ‘conscious venture’, ‘spirit at work’ and ‘coping strategies’. Each theme had 2-3 subthemes, populated by 14-23 statements.

**Conclusion:**

Expatriate faculty members described antecedents for their motivations at work. They shared their experiences regarding job adjustments, work environment and professional commitment.

## INTRODUCTION

Academic expatriation is an age old phenomenon. University scholars have travelled far and wide across the globe since medieval times.^1^ Experiences of academic expatriates are different from academics employed in their home countries as the former are prone to cognitive overload in a foreign work environment due to cultural differences and missed comforts of home.^2^

The reasons for expatriation in university teachers are directly related to their work outcomes and job satisfaction.^3^ Those driven by ‘explorer motivation’ experience greater job satisfaction as they tend to value the cross cultural interactions and thus progress smoothly through work adjustments.^3^,^4^ On the other hand, academic expatriates who are isolated from host culture usually operate as ‘tightrope walkers’, being avidly conscious of the risks of expatriation and precariousness of their job situation.^4^ Lucrative benefits that improve an expatriate’s lifestyle are the defining factors in individualized experiences of the academic expatriates. This compels most of them to stick to their positions irrespective of organizational climate and culture.^5^

A large number of academic expatriates are employed at universities in Saudi Arabia. The kingdom witnessed enormous development in the higher educational sector with phenomenal physical expansion of universities incorporating a large number of expatriate faculty.^6^ In recent years, the country’s Saudization program has picked momentum. It requires measures to reverse and minimize reliance on expatriates, which subsequently reduced the number of new expatriate employments and affected several job aspects of existing expatriate employees, mainly hired in the oil-boom years.^7^ The purpose of this research was to explore the experiences of expatriate university teachers at a health science university in Saudi Arabia.

## METHODS

The study had a basic qualitative descriptive design. Ethical approval was granted by the IRB in King Saud Bin Abdulaziz University for Health Sciences (IRBC/1538/19). Snowball sampling was used to recruit participants from different colleges in university (X). Thematic saturation was attained after thirteen interviews. In-depth face to face interviews were conducted in 2019 from 12 September to 20 October, using semi structured open ended questionnaire, following informed consent. The interviewees were informed about the project beforehand, and rapport was established before and during the interviews. They were approached with an open and curious attitude and genuine interest was expressed in their point of view. Interview lasted from 30-45 minutes. Interviews were conducted by NB at venue selected by the participants, audio taped and transcribed verbatim. NB maintained reflexive journal for bracketing. Member checking occurred with the interviewees. Transcribed Interviews were allocated numbers. Participant identities were concealed and pseudonyms were used in data reporting.

For data analysis, accuracy of the transcripts was matched to the audio recordings. The inductive approach proposed by Graneheim and Lundman (2004) was employed for manually analyzing the data, which comprises of four stages.^8^ Coding was done by NB and HAN. At stage one, evident experiences of the participants, particularly related to academic expatriation were found. This was done by thoroughly reading the transcripts several times (body of text). At stage two, significant similar statements that comprised of words, phrases, sentences and clauses were highlighted and selected (meaning unit). In the third stage of data analysis, categorization and summarization of the significant statements took place (condensed meaning unit). In the final stage, the summarized content was conceptualized to yield themes and subthemes (coded units).

The standards of credibility, dependability, transferability and confirmability proposed by Lincoln and Guba (1985) were applied for trustworthiness and rigor.^9^ For credibility of the results, the content underlying each one of the three themes was compared and cross checked with the feedback of the interviewees. To establish dependability, the researchers underwent mutual inspection and discussion, in pursuit of meaning and relevancy along with two qualitative study design experts until a joint agreement on conclusion was reached. To account for transferability, in-depth and rich description was provided. Confirmability was attained by an audit trail conducted on the exploratory process and initial raw data.

## RESULTS

All participants were non-westerners, full time PhD faculty members with (mean age 48±9.1 years). Ten out of thirteen participants were non-Arabic speakers. Eight participants were female and five were male. Sample comprised of two associate professors and eleven assistant professors. Participant’s job duration in the kingdom was within four to thirteen years.

Data analysis resulted in three themes namely ‘conscious venture’, ‘spirit at work’ and ‘coping strategies’. Each theme had 2-3 subthemes (Table-I). Each subtheme was populated with 14-23 statements.

**Table-I:** Themes and subthemes.

Themes	Subthemes
Conscious Venture	Work adjustments
	Cost of expatriation
Spirit At Work	Job insecurity
	Organizational climate
Coping Strategies	Strategic disengagement
	Purposeful bonds
	Contingency plan

### Conscious venture

The first theme was ‘conscious venture’ with two subthemes, ‘work adjustments’ and ‘cost of expatriation’. The participants described struggles made to adapt into a foreign work environment. ‘Work adjustments’ required acquisition of skills in order to integrate effectively into the new system. Efforts were spared to embrace socio-cultural diversity. Participants spoke of their struggles to overcome the language barriers, whilst other relayed on their pleasant learning experiences in a multicultural work environment.

*‘It’s not the course content but the classroom interactions, which are different. Most Saudi students have impeccable memorizing skills. Once I grasped how they learned, I had to revise my teaching techniques. It was initially difficult, but worth the efforts.’*(sharehan)

*‘I realized that these students learn differently and are accustomed to a different kind of learning environment. I started working on it, my class performance noticeably improved’*(Judy)

‘*I eventually grasped that written communication skills are vital in order to not get lost in translation. I learned that the hard way, but good that I did’* (Sarah)

*‘I am totally lost at some faculty meetings when the conversation breaks into pure Arabic.’* (Kim)

*‘Sometimes I feel sad when I see my advisers struggling to find the right words in English…. I try to improve my Arabic vocabulary to overcome such barriers.’*(Tania)

*‘Interactions with people from different backgrounds allow you to learn a lot. The different perspectives…problem solving skills…different approaches, all is very interesting and very different… you can explore like an adventurer who roams across different destinations.’* (Stan)

The study participants endured the ‘cost of expatriation’ and shared their experiences in context of how it affected their lives, families, relationships as well as career prospects at home.

*‘I sent both my sons to stay with my parents. Yes, it is hard! Expatriate children don’t have university education opportunities in Saudi Arabia….. My kids have a good education and I have better social entitlement in my family now as I am in a position to lend support to them, all thanks to this job.’* (Samah)

*‘I sent my daughters back with their mother, very tough times ever since. The girls always say they need me with them there. But it’s for the greater good for us all.’*(James)

*‘I get blamed for all that came my way because I chose this job abroad and moved with my young sons, it was the beginning of a strained relationship with my husband (employed back home), who was so thrilled and supportive initially.’*(Sahar)

*‘I could not take care of my father in his last days; I could not even make it in time in his last hours.’*(Sam)

*‘I am way past the age limit for applying for a pensionable government position in xxxx [country]. No more opportunities for me there.’*(Jack)

### Spirit at work

The second main theme was ‘spirit at work’. The participants spoke of several factors that affected their ‘spirit at work’ in relation to the ‘job insecurity’ and ‘organizational climate’. The participants reiterated that they were worried by the prospect of losing their jobs any time. This often led to fierce competition. They went the extra mile to please their bosses.

*‘I feel like hanging by the thread sometimes. I push myself at work to perform better, no matter what my personal battles are. We are driven by a constant urge to shine in front of our bosses and it gets exhausting.’*(Kim)

*‘You cannot afford to perform poorly….. You have already left several opportunities behind.’*(Tania)

*‘We never lose sight of the shark in the tank, the job insecurity is real. Although it gets stressful at times, but it also propels you towards hard work in order to survive, there is a visible struggle among expats trying constantly to prove their efficiency.’* (Stan)

Speaking of the organizational climate, notions of discrimination, incivility and perceived injustice were hinted in the dialogues of the participants, who expressed that it affected their commitment and performance.

*‘It’s the color of your passport that defines your salary. One day I discovered that two expatriate lecturers earn a lot more than me [assistant professor]. I sense a shift in my motivation level ever since’*.(Henry)

*‘You may sometimes encounter covert incivility, superiority complex and racial friction from other expats. It may take quite some time before you feel accepted.’*(Samah)

*‘Foreign qualified Saudi returnee scholars often say that their due positions are occupied by ‘others’, and to be frank, it is indeed the case. Not all, but a select few, consider themselves indispensable and more entitled for all perks and benefits.’*(Sahar)

*‘The mindset that you can pay to get the service still exists, especially towards people originating from the subcontinent.’* (Andrew)

*‘The administration makes visible efforts that we do not feel discriminated at any point; they go several lengths to be fair, supportive and motivating. Sometimes it gets very obvious that it’s not easy for them. I would rather back down from a conflict point than be in such a situation. All of it eventually gets to you’*(Judy)

### Coping strategies

The third main theme was ‘coping strategies’ with three subthemes namely, ‘strategic disengagement’, ‘purposeful bonds’ and ‘contingency plan’. The participants relayed on that they occasionally allow themselves to temporarily withdraw, in order to revitalize and boost their momentum. 

*‘I just need a trip to xxxxx [home country] when it gets overwhelming. Truth is that, on most return trips, I am overly grateful for my job, after being reminded why I chose this job in the first place [Laughing].’*(Henry)

*‘Sometimes I shift my focus towards my own career growth, opt for an international conference, complete long-ignored research projects or connect with intellectual influencers. You know short escapes from the regular grind. We often recommend this to each other [Expatriates], because it’s therapeutic’*(Sahar)

*‘I feel that I am strongly committed to the honesty thing in my profession and my bond with my students, but not as much to the university.’* (Jack)

*‘What keeps me going are my students. I practically look forward for my interactions with them and can easily avert eyes from the rest.’*(Sam)

Another coping strategy practiced by the participants owing to their personal experiences is ‘purposeful bonds’ with people at work.

*‘Expatriates with same nationalities often operate in groups. They rely on each other for swift information exchange and support each other blindly, without genuinely liking each other.’*(Kim)

*‘You will see that some expatriates will always have ‘friends’ in important places, sometimes more like a network. It a survival game and they [expatriates] go great lengths for it.’*(Sharehan)

Every participant nurtured a personalized ‘contingency plan’ to secure their careers in future.

*‘The most repeated conversation that we have among ourselves is about having a Plan B. Many expatriates came here on Foreign Service leaves and they maintain strong ties with helpful contact persons at home. Now this international experience is an added bonus’*(Sarah)

*‘As a university faculty member in Saudi Arabia, you have a highly paid and prestigious job and you are able to save a large chunk from your salary without brutal tax cuts. But, you need to prepare an alternative plan, in order to not fall flat on your face. You need to cash your foreign experience when you still can!’*(Henry)

## DISCUSSION

The purpose of this study was to explore experiences of expatriate university faculty in Saudi Arabia. The three main themes following data analysis were ‘conscious venture’, ‘spirit at work’ and ‘coping strategies’.

The participants described that they were mindful of the efforts required to adjust in a foreign work environment with socio-cultural and linguistic barriers. Job adjustment required acquisition of techniques which complied with the learning process of the local students. Adaptations to local context and transforming current practice is necessary for even formerly acclaimed skilled expatriate academic.^10^ Academic expatriates are thus more attuned to cultural differences by gaining valuable insights into new teaching practices and processes.^11^ Socio-cultural diversity enticed some participants whereas others felt challenged by language barriers after being perplexed at meetings with students and faculty. They focused on ways to overcome such barriers e.g. improving Arabic vocabulary, preferring written communications etc. This demonstrates mental resilience, patience and congeniality, which are frequently noted traits in expatriate academics.^10^,^12^ The participants expressed that they knowingly paid the price for expatriation because of the associated benefits. They handled their personal battles whilst consciously reminding themselves of the greater good. They risked ties with close family members and career prospects at home. For them, it was not entirely a delightful situation. Pursuit of financial gains and stability through expatriation often causes regrets and dissatisfaction in academics as they eventually get tied to the ‘golden handcuffs’, which are not easily shaken off.^4^

The second theme was ‘spirit at work’ based on how the participants experienced job insecurity and organizational climate. Most of them were practically haunted by the prospects of relocating after losing their jobs. It propelled them to work harder and outshine others in efficient conduct, leading to mental exhaustion and cognitive overload. Such career risks drain energy in academic expatriates.^13^ In terms of organizational climate, some participants felt belittled because of their country of origin. Extant literature endorses that job positions and benefits are often linked with demographics of expatriate academics.^1^,^12^ Even though the study participants openly admired the efforts made by the Saudi administration to maintain fairness and compatibility in the work place, it did not prevent them from being affected by subtle remarks made by other expatriates or Saudi colleagues. They were apprehensive from the occasional blame of ‘stealing jobs’ from the locals. They mentally compared their status with fellow expatriates who enjoyed better salaries owing of their nationalities and Saudi colleagues who operated without insecurities. These findings affirm that individuals resort to stereotyping in foreign work environment to mitigate mental burden of intercultural interactions, which interferes with their thought process and impacts emotional engagement at work.^14^ Statements made by the study participants reflect perceptions of self-esteem, emotional steadiness and internal locus of control, which are predictive of job satisfaction and performance in academic expatriates.^3^

The third theme was ‘coping strategies’ that allowed us to delve into the inconspicuous defensive measures strategically taken up by academic expatriates in attempts to self-sustain and thrive. The study participants often used mental and practical strategies of disengagement from workplace. They expressed that they allowed themselves to get attached to their students who were a bigger source of joy and contentment for them than the work environment. They valued their interactions with the students and basked in essence of being true to their profession. This phenomenon is often observed in expatriate teachers who cherish their commitment to the students more than the organization itself, by vehemently supporting their growth and learning.^12^,^15^ Strategic disengagement also involved escaping into an environment that allowed them to revitalize and motivate themselves, which sometimes meant tending to long term career prospects or a visit to their homelands, the latter being a coping strategy frequented by expatriates.^16^ The study participants maintained purposeful social ties in order to gain necessary and timely information as well as support in their work place. This is in line with a previous study showing that academic expatriates strategically formed friendships with other expatriates as well as host nationals to proficiently encounter difficulties and hurdles.^17^ They were also mentally prepared for a fresh start. They weighed their options, tested waters for future prospects and counted their benefits as an expatriate (e.g. international experience) in attempts to assuage notions of insecurity. The study participants expressed hope that their international experience and job contacts will be fruitful in future job pursuits. Extant literature shows similar concerns from academic expatriates on perceived value of international experience as they kept their eyes open for new opportunities.^3^,^17^

### Limitations of the study

It is a single institute study and excludes western expatriate faculty members. Data source triangulation is recommended for future studies.

## CONCLUSION

This article offers an insight into the conscious ventures of expatriate faculty members primarily comprising of adjustments to a set of different job expectations and adaptations within a unique socio-cultural context. Diverse factors affected their job spirit. They employed strategies to overcome hurdles and secure their academic career.

### Authors’ Contribution

**UA** did data evaluation, manuscript writing and critical revision ensuring integrity of the study.

**NB** conceived research idea, did interviews, manuscript writing, data analysis and evaluation, and ensured accuracy of data.

**HAN** did literature review, data analysis, manuscript writing, ensured accuracy of data and critical revision.
